# Bioinspired liquid metal-spidroin (LMS) stripe microneedle actuators

**DOI:** 10.1016/j.mtbio.2026.103662

**Published:** 2026-09-10

**Authors:** Xin Li, Min Xue, Kaiwen Zhou, Ran Xu, Hao Ren, Bingbing Gao, Chwee Teck Lim, Feng Liu

**Affiliations:** a College of Biotechnology and Pharmaceutical Engineering, School of Pharmaceutical Sciences, Nanjing Tech University, Nanjing, 211816, China; b Institute for Health Innovation and Technology (iHealthtech), National University of Singapore, Singapore, 117599, Singapore; c Department of Biomedical Engineering, National University of Singapore, 117583, Singapore; d Mechanobiology Institute, National University of Singapore, 117411, Singapore; e Research Center of Allergy & Immunology, Shenzhen University School of Medicine, Shenzhen, China

**Keywords:** Microneedle actuator, Liquid metal-spidroin composite, Photothermal soft robotics, Triboelectric nanogenerator, Smart wound dressing

## Abstract

Intelligent wound dressings have demonstrated significant clinical value. Among which flexible actuators dressings hold promise for real-time monitoring and on-demand treatment adjustment. Herein, we develop a novel bioinspired liquid metal-spidroin (LMS) stripe microneedle (MN) actuators for multifunctional wound management. The LMS system consists of recombinant spider silk proteins (spidroin) matrices embedded with gallium-based liquid metal droplets and patterned into asymmetric microneedle stripes by casting and programmed cutting. Benefiting from selaginella-inspired layouts, LMS composites exhibit near-infrared-triggered bending and coiling, while morphology-encoded microchannels guide interstitial fluid along predefined routes without external pumps. The liquid metal core also functions as a deformable electrode and triboelectric nanogenerator, enabling energy harvesting and self-sensing during microneedle deformation. In vitro and in vivo experiments demonstrate that epidermal growth factor-loaded LMS microneedle bandages conformally wrap cutaneous defects and significantly accelerate wound closure and re-epithelialization compared with control dressings. Histological analysis reveals no evident systemic toxicity, indicating acceptable biosafety. These findings suggest that LMS stripe microneedle actuators provide a promising platform for next-generation intelligent wound dressings.

## Graphical abstract

**Figure:**
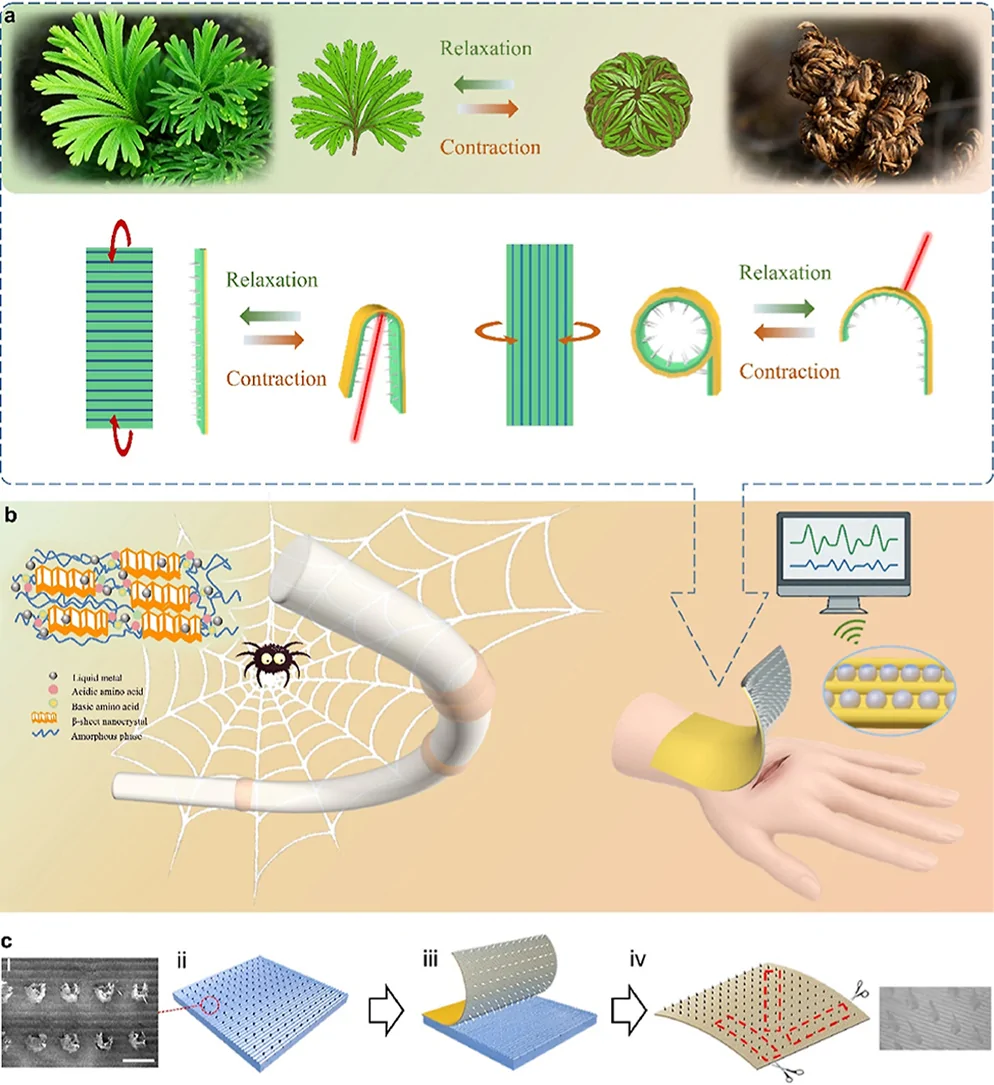

## 1 Introduction

Chronic skin wounds are the persistent defects of cutaneous tissue, which can lead to prolonged inflammation, infection, disability, and even limb loss, and impose a heavy healthcare and economic burden. Due to the urgent clinical need for effective and intelligent wound management, great scientific attention has been focused on developing advanced dressings that can regulate the wound microenvironment, promote tissue regeneration, and provide real-time feedback on healing status. Various approaches, such as hydrogel and film dressings, porous foams, microneedle patches, and smart bandages integrating sensors and drug reservoirs, have been developed to protect wounds, deliver therapeutic agents, and monitor biochemical or mechanical cues [[1], [2], [3], [4], [5], [6]]. Generally, these wound care systems can maintain a moist environment, reduce infection risk, and accelerate re-epithelialization, serving as essential tools for the treatment of acute and chronic wounds. However, most of the available dressings are planar, passive, and mechanically mismatched with dynamic skin, which may result in poor conformal contact, insufficient control over exudate transport, and limited ability to respond to body motion or evolving wound geometry. In addition, existing platforms often treat actuation, adhesion, sensing, and therapy as separate modules, which tends to increase device complexity and hinder miniaturization and long-term stability at the tissue interface. Thus, it is imperative to develop a new class of soft actuator–based wound dressings that can actively morph to match irregular defects, anchor securely via minimally invasive microneedles, guide biofluids without external pumps, and coordinate sensing and therapeutic functions within a single integrated system [7,8].

**Fig. 1:**
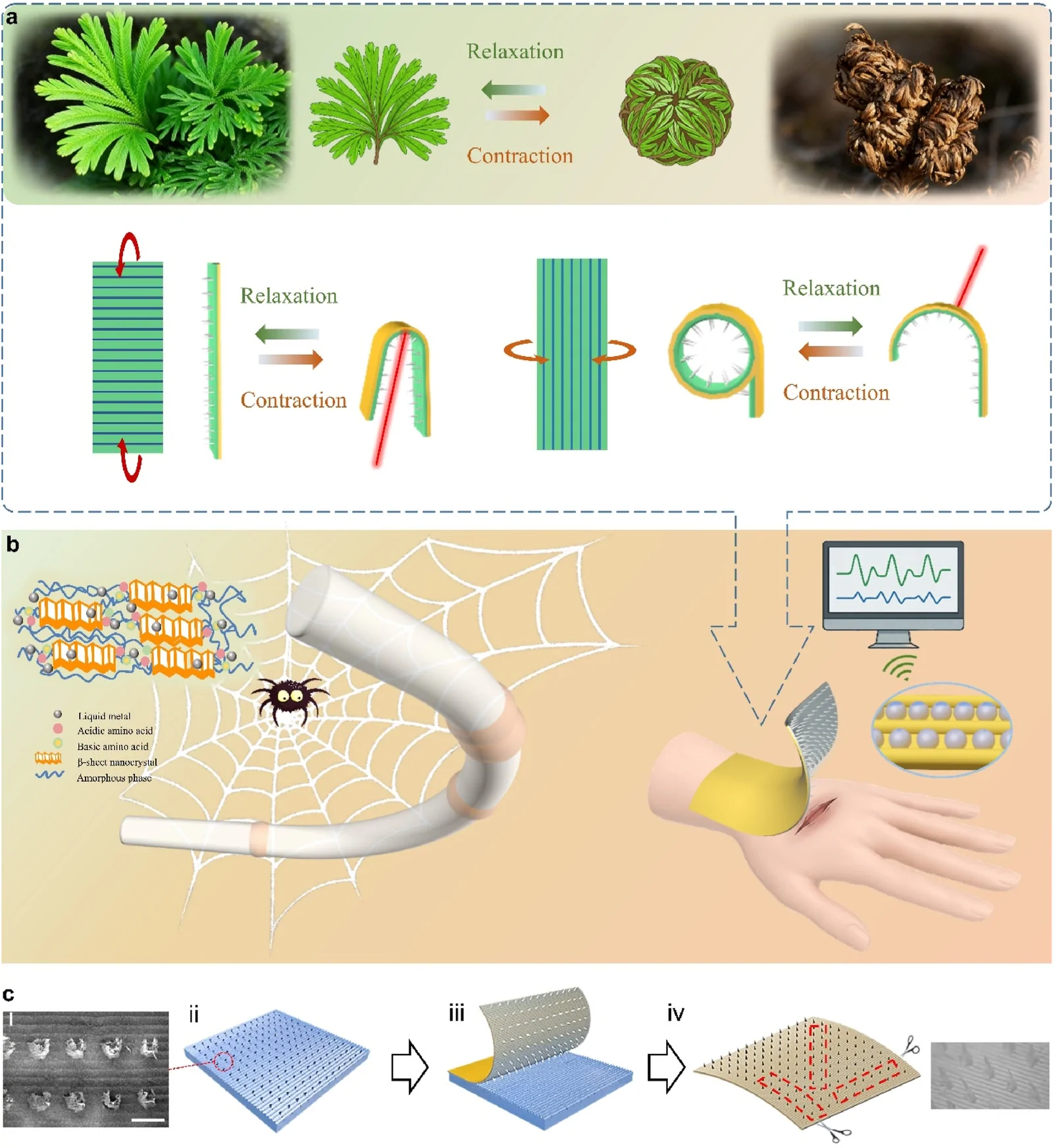
Bioinspired design and fabrication of the LMS MN Actuator. (a) Morphological transition of Selaginella leaflets between relaxed and contracted states, demonstrating the desiccation-induced curling mechanism. (b) Schematic illustration of the LMS MN Actuator, in which the inner layer consists of amorphous-region-modified artificial Spidroin integrated with liquid metal, enabling photothermal actuation and motion sensing capabilities. (c) Development of the LMS MN Actuator. Altering the angle of stripes through different cutting methods. (Scale bar: 200 μm)

In this work, inspired by the anisotropic local deformation mechanism guided by the leaf veins of *Selaginella tamariscina* leaves, we propose a novel bioinspired liquid-metal-spidroin (LMS) stripe microneedle actuator platform for multifunctional, biointegrated wound management(Fig. 1). Soft actuators are deformable devices that convert external stimuli into programmable shape changes, which have recently become a research hotspot in soft robotics and wearable electronics due to their high compliance, lightweight nature, and ability to operate in confined or delicate environments [[9], [10], [11], [12]]. Generally, soft actuators tailored with diverse actuation modes, such as photothermal, magnetic, and thermal stimuli, and implemented in architectures including bilayer films, kirigami sheets, and origami-inspired structures, have been developed to achieve bending, twisting, rolling, and gripping motions [[13], [14], [15], [16], [17], [18], [19], [20], [21], [22]]. Liquid-metal–based composites, combining highly deformable metallic droplets with elastomeric or hydrogel matrices, further extend this toolbox by offering excellent photothermal conversion, electrical conductivity, and mechanical robustness [23,24], and have been explored for actuation, flexible conductors, and energy harvesters [25,26]. Microneedle arrays, composed of microscale needles arranged on flexible substrates, provide minimally invasive access to interstitial fluid and deep skin layers, enabling enhanced drug delivery, fluid sampling, and the integration of sensors or therapeutic cargos. However, these device classes address complementary but distinct functional needs. Microneedle dressings are primarily designed for drug delivery or fluid sampling, whereas soft actuators and liquid-metal composites generally focus on programmable deformation, conductive sensing, or energy harvesting. Consequently, integrating secure microneedle-mediated tissue anchoring, stimulus-responsive shape morphing, directional pump-free biofluid transport, and stable electrical signal generation within a single wound-interface platform remains challenging. This integration gap motivates the unified material and structural design of the LMS platform described below [[27], [28], [29], [30]].

Here, we aim to develop an advanced active treatment platform specifically for chronic wound management [[31], [32], [33], [34]]. We present a unified liquid metal-spidroin (LMS) strip microneedle actuator. The core theoretical innovation of our study lies in the unprecedented spatial integration of deterministic stripe angle programming with a functional microneedle topology [35]. By applying photothermal soft robotic actuation technology clinically, the dressing is programmed to actively contract upon near-infrared (NIR) irradiation, mechanically bringing the wound edges together to accelerate macroscopic healing. Simultaneously, this engineered microneedle topology possesses an important dual function: it provides robust tissue interlocking for continuous, diffuse delivery of human epidermal growth factor (hEGF); furthermore, the morphology-encoded microchannels formed by the integrated stripes and microneedles control wound exudate through self-driven, pump-free capillary action. This sophisticated fluid management system, combined with spidroin/biaxially oriented polypropylene (BOPP) film encapsulation, structurally protects the integrated liquid metal network. Therefore, the liquid metal core reliably functions as a self-powered biomechanical sensor, continuously monitoring patient mobility and dressing integrity even in physiological settings. Ultimately, these material and robotic innovations are not demonstrated independently but synergistically to build a next-generation intelligent wound care system.

## 2 Results and discussion

### 2.1 Design of the LMS microneedle (MN) actuators

**Fig. 2:**
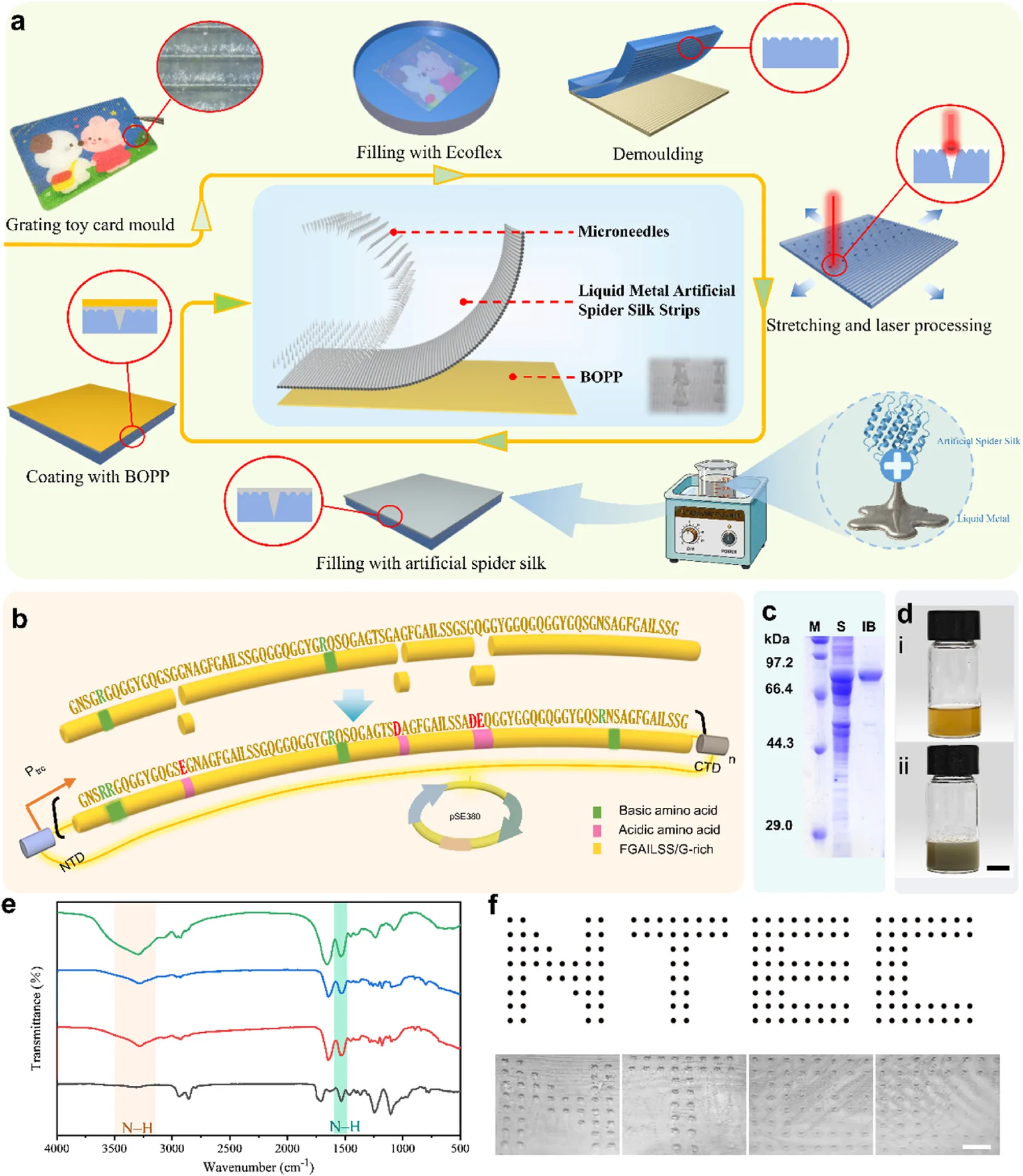
Amorphous-region modification of artificial spidroins (ARM) and construction of LMS MN actuators.(a) Step-by-step fabrication of the LMS MN actuator: grating-card mold casting with Ecoflex, demolding, BOPP coating, filling with artificial Spidroin, followed by stretching and laser patterning to obtain microneedle arrays and LMS strips.(b) Concept of amorphous-region modification to enable high-level expression, direct purification, facile molding, and enhanced mechanical/electrical properties. Shown are the primary sequence of the core region of the artificial amyloid spidroin (Amy-3rep) and an ARM variant (Amy-ab-1), together with the genetic constructs used for ARM production. (c) SDS-PAGE analysis of engineered ARM. Lane designations: M, molecular-weight marker; S, soluble fraction; IB, inclusion bodies.(d) Morphology of the ARM dissolved in hexafluoro-2-propanol (HFIP) (i) and after addition of liquid metal (LM) (ii).(e) Infrared characterization of LMS MN actuators with three LM loadings (top to bottom) and a LM-free control.(f) Programmable assembly into designed patterns “N”, “T”, “E”, and “C”. (Scale bar: 15 mm)

Inspired by the efficient curling of *Selaginella* leaves under drying conditions to minimize water loss during environmental stress, we translated this biomimetic strategy into a liquid metal-Spidroin microneedle (LMS MN) actuator. This design achieves programmable deformation by mimicking the leaf's behavior around the vein bending axis. In our design, liquid metal (LM) droplets embedded in amorphous region-modified Spidroin (ARM) enable a targeted photothermal response. Device-level programmability is achieved through the initial design rather than post-design adjustments. As summarized in the step-by-step workflow (Fig. 2a), we extract stripe templates from commercial grating cards to cast an Ecoflex elastomer negative mold. A hexafluoroisopropanol (HFIP) solution containing ARM and LM is filled into the cavity. After solvent evaporation, a lightweight biaxially stretched polypropylene (BOPP) barrier layer is laminated, followed by uniaxial stretching to introduce pre-strain and expose the stripe-guided bendable axes. Finally, stretch-assisted laser micromachining transforms these templates into microneedle arrays and actuator stripes. This approach fixes the deformation axis in-plane while allowing the chosen cutting angle to determine the out-of-plane stripe angle (Fig. 1c). Under illumination, the temperature preferentially increases along the stripe-induced flexible direction, driving predictable bending, while the electrical pathways enable synchronous motion sensing.

To optimize the core materials, we engineered an artificial spidroin (ARM) to address the bottleneck limiting recombinant spider silk production while improving its mechanical and electrical properties. The core domain consists of alternating β-sheet crystalline and amorphous fragments; while traditional research focuses on crystalline structures, amorphous fragments govern stretchability, assembly, and energy dissipation. By introducing acidic and basic residues and reducing the proportion of glycine in the amorphous segments, we altered the protein's polarity. This sequence editing (Fig. 2b) prevents hydrophobic aggregation and enables direct, cost-effective purification via simple thermal cycling without affinity tags. Quantitative analysis further demonstrates this improved manufacturability: the engineered ARM spidroin achieves a highly impressive volumetric yield of 803.33 mg/L (calculated as the mass of purified lyophilized powder per volume of culture medium), which successfully overcomes the low-yield bottlenecks typical of traditional recombinant spidroin production. Furthermore, interlayer salt bridges and ion pairs in the amorphous matrix enhance molecular cohesion, stabilizing the β-sheet nanocrystal network during deformation and enhancing load-bearing capacity. SDS-PAGE validates the expression and solubility, distinguishing molecular weight standards (M), soluble components (S), and inclusion body components (IB) to support large-scale direct purification (Fig. 2c). The resulting LM-containing HFIP dispersion highlights a uniform appearance and stable dispersion, crucial for defect-free curing (Fig. 2d). The resulting LM-containing HFIP dispersion exhibited a uniform appearance and remained stable during processing (Fig. 2d). To further investigate the spatial distribution of the LM droplets within the cured composite, SEM–EDS elemental mapping was performed (Fig. S17). The homogeneous distribution of the Ga signal throughout the analyzed region confirmed that the liquid metal droplets were uniformly dispersed within the spidroin matrix. Consequently, the modified silk fibroin withstands axial loads of tens of Newtons—exhibiting a tensile strength over an order of magnitude higher than unmodified controls [36]—and generates enhanced triboelectric peak open-circuit voltages approaching tens of volts.

The final device integrates the geometry and material layers into a scalable production line, producing actuators with well-defined anisotropy, efficient photothermal conversion, and stable interfaces. Fourier transform infrared spectroscopy (FTIR) (Fig. 2e) shows that changing the loading of the LM systematically alters the characteristic absorption bands, reflecting changes in hydrogen bond and salt bridge densities. Mechanical screening showed that the composite containing 10 wt% LM retained a maximum stress comparable to that of the LM-free spidroin, whereas further increases in LM content markedly compromised structural integrity (Fig. S15). The 75 wt% LM sample could not maintain sufficient structural integrity for reliable mechanical testing. Although increasing the LM content may facilitate conductive-pathway formation and photothermal heating, excessive loading can disrupt matrix continuity and reduce overall actuator stability. Therefore, 10 wt% LM was selected as a practical balance between mechanical integrity and LM-enabled functionality. Geometric instructions can be reliably transmitted to the assembly stage without deformation, enabling programmable macroscopic patterns such as “N,” “T,” “E,” and “C” layouts (Fig. 2f). While protein engineering at the molecular level provides the mechanical strength required for stable actuation, the specific macroscopic deformation patterns are deterministically encoded by geometric stripe programming and cutting angles. Under near-infrared irradiation, the LM coating acts as a localized heat source, while the protein network and biaxially oriented polypropylene (BOPP) laminate maintain shape fidelity and prevent moisture-induced warping. Simultaneously, the LM network forms deformable conductive pathways for resistance-based motion readout. With the MN interface, this composite material can be firmly attached to soft tissue without bulky fasteners, laying the foundation for pump-free biofluid transport and the operation of triboelectric nanogenerators.

### 2.2 Photothermal actuation and programmable locomotion of LMS MN actuators

**Fig. 3:**
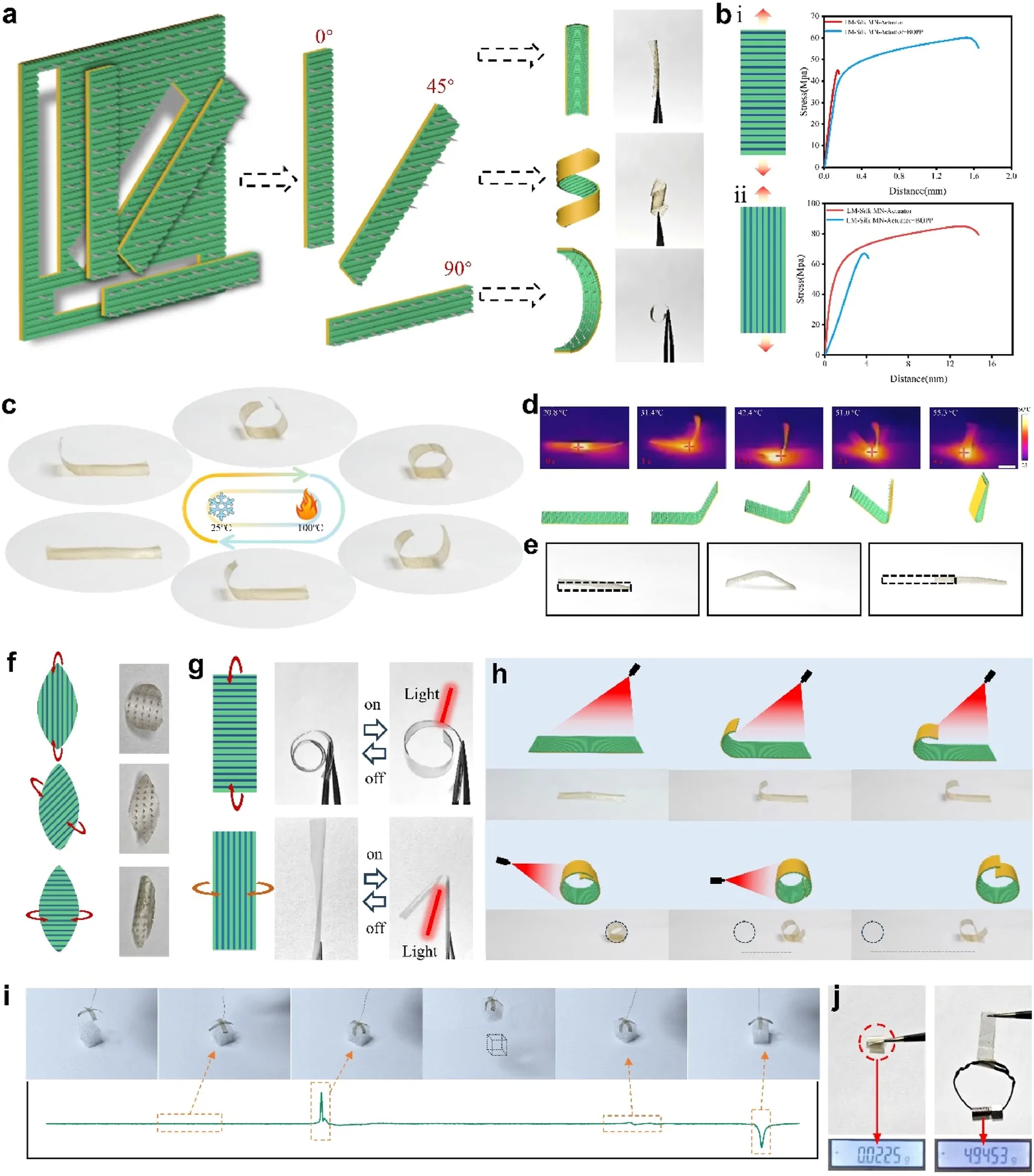
Photothermal actuation and programmable locomotion of LMS MN actuators. (a) Three cutting angles (0°, 45°, 90°) program distinct morphologies—bending, twisting, and coiling. (b) Mechanical testing setup and stress-displacement responses: (i) stripes perpendicular to the loading axis; (ii) stripes parallel to the loading axis; comparison between bare actuators and BOPP-backed actuators. (c) Reversible shape changes of the actuator with temperature.(d) Infrared thermograms showing temperature evolution under near-infrared (NIR) illumination.(e) Crawling locomotion realized by the photothermally driven soft robot.(f) Programmable customization via the three cutting angles to generate diverse shapes.(g) Deformation modes governed by stripe orientation relative to the long axis.(h) Rolling locomotion under NIR irradiation.(i) PFA-assembled soft robotic gripper: grasp-lift-release sequence with synchronous voltage response trace.(j) Mass measurements of the LMS MN actuator and its carried load on an electronic balance.

LMS microneedle (MN) actuators spontaneously curl along the embedded artificial Spidroin stripes, acquiring a programmable initial shape. By cutting the striped sheets at 0° (parallel), 45° (oblique), and 90°(perpendicular) to the stripe direction, we obtained vertical, torsional, and curled actuators (Fig. 3a). This demonstrates the cutting angle as a robust handle to program actuator morphology.

To quantify the mechanical anisotropy behind this geometric coding, we conducted uniaxial tensile tests on LMS MN actuators cut along the 90° and 0° directions, testing with and without a BOPP backing (Fig. 3b). In the 90° configuration (stripes perpendicular to the load direction, Fig. 3bi), the bare actuator fractured at approximately 45 MPa of stress and 0.2 mm of displacement. After laminating a thin BOPP film, the peak stress increased to 55–60 MPa, and the fracture displacement increased to approximately 1.8 mm, significantly improving toughness and energy dissipation. In the 0° configuration (Fig. 3bii), stiffness and ductility were further improved: the bare actuator reached 65–70 MPa of stress at a displacement of 3.5–4.0 mm, while the BOPP-backed actuator withstood approximately 90 MPa of stress and elongated to 15–16 mm before fracture. For reference, traditional commercial wound dressings made of hydrogel and silicone foam typically have a tensile strength of less than 5 MPa, making them prone to tearing under high physiological stress. In contrast, the LMS actuator with a BOPP backing possesses extremely high strength and good ductility, ensuring superior clinical durability. This characteristic allows the dressing to easily withstand repeated multidirectional deformations of dynamic human skin without mechanical failure, thus ensuring the integrity of the wound's physical barrier and the long-term reliability of the internal electrical sensing network. This mechanical anisotropy directly translates into programmable actuation behavior. Under NIR irradiation, the pre-programmed LMS MN actuators exhibited reversible bending, twisting, and curling (Fig. 3c). Time-resolved infrared images showed continuous shape deformation as the actuator temperature increased from 28.5 to 55.3 °C within 4 s (Fig. 3d and Fig. S11b). The bending angle increased with temperature, with an actuation speed of 32° s^−1^ (Fig. S11c and f). Moreover, reproducible bending and recovery were maintained over 40 consecutive NIR ON/OFF cycles (Fig. S11e), demonstrating the rapid and stable photothermal response of the actuator.

Transforming this photothermal bending into directional locomotion, we operated the LMS MN actuators as soft robots. Cyclic NIR irradiation generated asymmetric bending-straightening cycles. This localized heating, combined with asymmetric substrate contact, produced stable, directed crawling with cumulative displacement and no noticeable mechanical degradation over multiple cycles (Fig. 3e).

This design strategy also enables biomimetic morphologies, such as a leaf-like soft robot (Fig. 3f). Shallow grooves act as compliant hinges, while raised Spidroin stripes serve as reinforcing “veins” defining the bending axes (Fig. 3g). By selecting specific cutting angles relative to these veins, the 3D configuration is deterministically programmed to achieve predictable, multistage deformation. Under NIR light, this structure can execute controlled rolling locomotion via repeated bending around an eccentric axis (Fig. 3h).

Furthermore, the LMS MN platform integrates tactile sensing and high load-bearing capabilities. A soft robotic gripper was developed (Fig. 3i), where grasping deformations generated analog electrical signals. These were converted into digital readouts, establishing a reliable, real-time correlation between the sensor output and the gripping state. Finally, load testing demonstrated that a single actuator could lift a payload 219 times its own weight (Fig. 3j S11), showcasing an exceptional force-to-weight ratio for robust, compliant applications.

### 2.3 Design of the LMS MN actuator hydrodynamic device

**Fig. 4:**
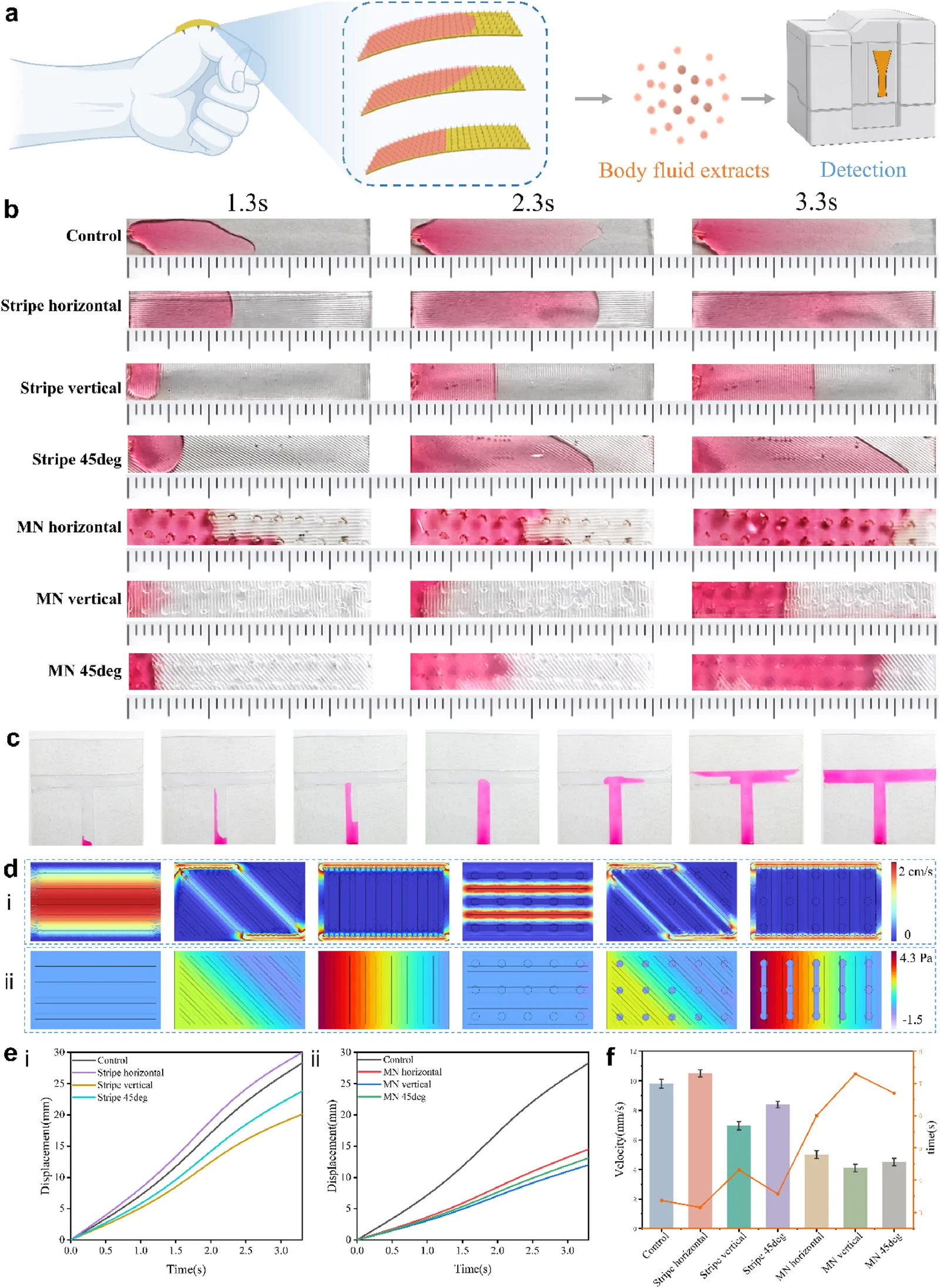
Pump-free biofluid transport guided by patterned LMS MN actuators.(a) Concept of morphology-encoded transfer using LMS actuators with stripe or microneedle (MN) patterns to steer body-fluid extracts without external pumps.(b) Time-lapse images of spontaneous flow in channels with different stripe orientations (horizontal, vertical, 45°) and with/without microneedles; rows include control (no pattern), stripe-only (0°, 90°, 45°), and MN-integrated channels with the same stripe orientations. (c) Schematic of pump-free flow within a T-shaped patterned channel.(d) COMSOL simulations for single-channel transport across stripe angles (0°, 90°, 45°), comparing stripe-only versus MN-integrated designs: (i) velocity fields; (ii) pressure distributions.(e) Travel distance versus time under pump-free conditions: (i) stripe-only channels; (ii) MN-integrated channels (each at 0°, 90°, 45°).(f) Summary of flow velocity and time to reach the distal end for all actuator channel types.

To extend morphology programming to pump-free fluid handling, we investigated capillary-driven transport in patterned LMS microneedle (MN) actuators. In this configuration, the LMS sheet functions as a morphology-encoded layer that steers liquid using only capillary pressure generated by its surface microstructures. As illustrated in Fig. 4a, shallow grooves, raised artificial Spidroin stripes, and superimposed MN arrays create a hierarchical capillary network. The grooves act as compliant channels, while raised features provide regions of enhanced curvature and preferential wetting. We evaluated this pump-free flow using time-lapse experiments in straight channels with flat (control), stripe-only, and MN-integrated patterns oriented at 0° (horizontal), 90° (vertical), and 45°. In stripe-only channels, 0° stripes guided the meniscus along low-resistance lanes, yielding the fastest, smoothest axial advancement. Vertical (90°) stripes introduced transverse ridges that repeatedly pinned and released the contact line, resulting in a staircase-like progression and lower average speed. Oblique (45°) stripes spread the fluid diagonally, slightly reducing net axial velocity. Adding MNs systematically changed the flow behavior. The MNs acted as dense capillary “hot spots” that rapidly wicked liquid, making the initial wetting more robust and stabilizing the advancing front against external disturbances. Consequently, horizontal MNs formed continuous rails, vertical MNs partially compensated for transverse resistance via added suction, and 45° MNs produced a strongly directed, laterally spreading front.

COMSOL simulations of the internal velocity and pressure distributions (Fig. 4d S7ab) clarify this morphology encoding. For stripe-only channels, 0° stripes produced a uniform axial velocity and a smooth pressure drop characteristic of efficient capillary flow. Rotating to 45° tilted the velocity field and skewed the pressure distribution, reflecting increased viscous dissipation. At 90°, the segmented velocity field and alternating pressure bands reflected successive pinning-depinning events, matching the experimental stepwise progression. Introducing MNs sharpened local pressure gradients and increased flow path tortuosity, enhancing initial imbibition but reducing net axial speed compared to stripe-only designs. Displacement-time curves (Fig. 4e) quantitatively reflect these trends. Within 3.2 s, 0° stripe channels reached ∼30 mm (surpassing the 26-27 mm control), while 45° and 90° channels advanced to ∼23-24 mm and ∼20-21 mm, respectively. For MN-integrated channels (Fig. 4eii), displacements ranged from 12 to 15 mm. This combination of enhanced wettability and increased drag yields slower, yet highly controlled and robust flow.

Fig. 4f summarizes average flow velocities and arrival times across all pattern types. Control and 0° stripe channels exhibited the highest velocities (9-11 mm s^−1^) and shortest arrival times (3-3.5 s), optimal for rapid fluid delivery or removal. Vertical and 45° stripes provided intermediate transport (7-9 mm s^−1^, 4-4.5 s). MN-integrated channels generated slower velocities (5-7 mm s^−1^) and longer arrival times (5.5-7.5 s) but offered superior meniscus stability and wicking robustness. Horizontal MN patterns offered the best speed-stability compromise, while vertical MNs yielded the most highly regulated transport. Ultimately, stripe orientation primarily tunes the capillary driving force versus geometric resistance, while MN integration homogenizes and stabilizes flow at the expense of axial velocity. By appropriately selecting the stripe angle and MN configuration, LMS MN actuators can be engineered to either rapidly wick wound exudate or deliver therapeutics in a sustained manner. Combined with their actuation and sensing capabilities, this microfluidic control positions LMS MN platforms as versatile bases for next-generation, pump-free programmable wound dressings.

### 2.4 Triboelectric performance of LMS MN actuator-based TENG

**Fig. 5:**
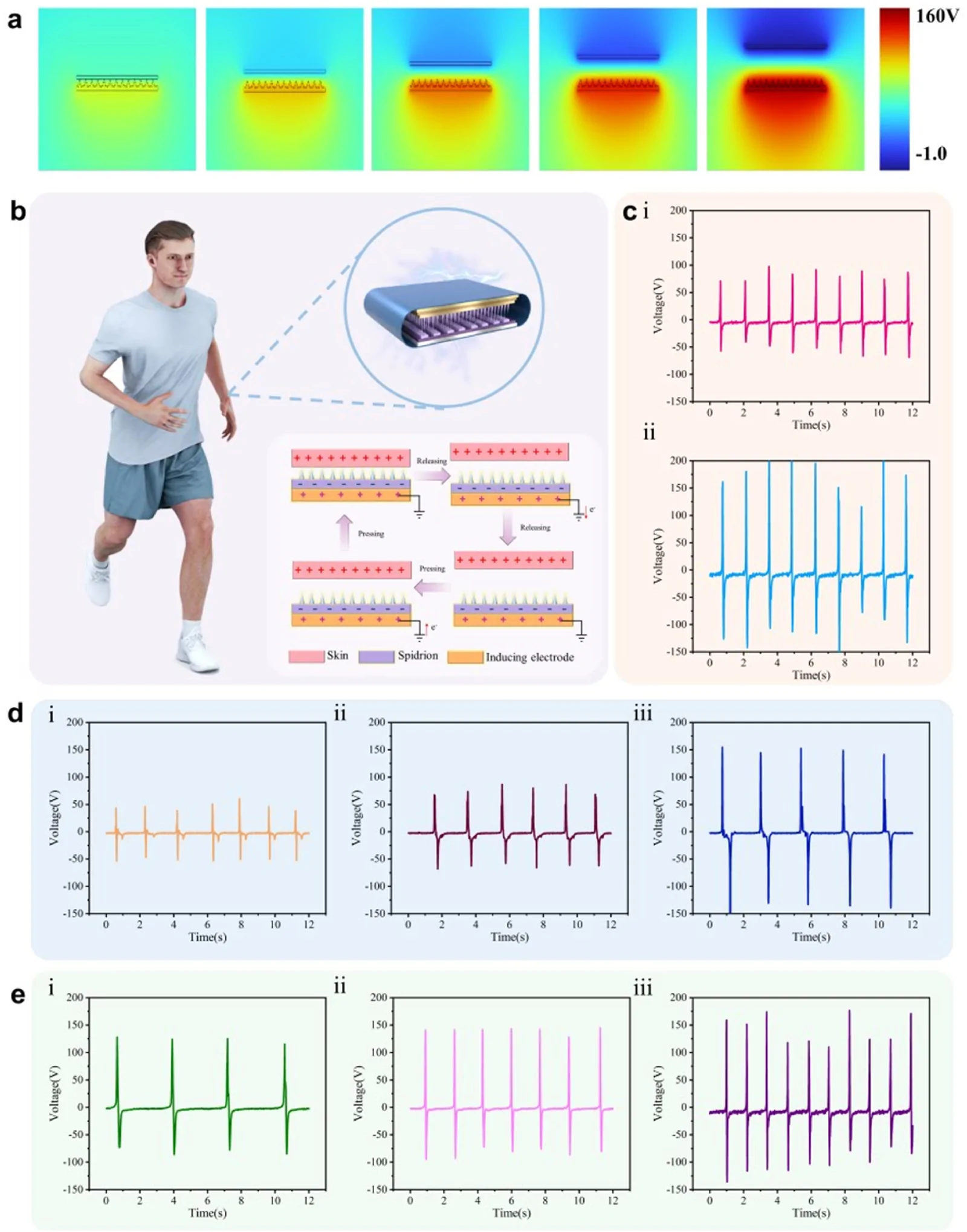
Triboelectric performance of LMS MN actuator-based TENG.(a) COMSOL-simulated potential distribution of the triboelectric microneedle unit (TPMN); scale bar: 500 μm. (b) Working principle of contact-separation triboelectric power generation driven by the LMS MN actuator. (c) Voltage outputs under identical conditions for (i) MN actuator without liquid metal and (ii) MN actuator with liquid metal. (d) Voltage output of the LMS MN actuator under friction at stripe orientations of 0° (i), 45° (ii), and 90° (iii).(e) Representative voltage waveforms under slow-to-fast rubbing conditions (i-iii).

By designing an LMS MN actuator with a triboelectric mechanism, efficient and adjustable electrical stimulation output can be achieved in localized wound areas. In practical wound applications, such electrical outputs are not intended for energy supply but for self-powered sensing, where signal variations can reflect deformation states, body motion, and dressing integrity at the skin interface. The LMS MN array, triboelectric negative electrode polymer film, and LM core serve as the high triboelectric positive electrode layer, high triboelectric negative electrode layer, and electrode layer, respectively. During periodic contact-separation processes, equal amounts of opposite triboelectric charges gradually accumulate on the LMS MN surface and the opposing triboelectric negative electrode layer, generating a time-varying potential difference on both sides. This potential difference causes charge redistribution in the LM electrode, thereby driving electrons to be directionally transported through an external circuit. COMSOL simulations of the potential distribution confirm the significant potential difference between the LMS MN tip and the dual surface during contact-separation. The high potential concentration at the microneedle tip and shank, with the high-potential region extending deeper into the surrounding medium, indicates that the microneedle geometry effectively focuses the electric field, and the continuous liquid metal core effectively collects the charge (Fig. 5a).

The charge transfer process can be described in detail as the contacting objects periodically approach and separate (Fig. 5b). In the initial contact state, the tight contact between the liquid metal microneedle surface and the layer of triboelectric negative charge leads to triboelectric charge transfer, resulting in a positive charge on the liquid metal side and a negative charge on the other. The charges on both sides are almost balanced, and there is no noticeable current flow in the external circuit. When the liquid metal microneedle actuator bends and the two surfaces begin to separate, a finite gap is formed, creating a potential difference between the liquid metal electrode and the reference electrode. In response, electrons flow from the liquid metal electrode to ground until the induced charge compensates for the triboelectric surface charge. When the actuator relaxes and the surfaces approach again, the potential polarity reverses, forcing electrons to flow back and generating a current in the opposite direction. This repeated contact-separation cycle produces an alternating voltage output between the terminals. Thanks to the high conductivity and uniform filling characteristics of the liquid metal network, the amount of charge transferred in each cycle increases significantly. Compared to a structurally similar but unfilled LM MN actuator, the LMS microneedle actuator exhibits a significantly higher open-circuit voltage and a more regular, symmetrical waveform under the same operating conditions (Fig. 5c). This enhancement indicates that the LMS structure not only strengthens triboelectric coupling at the interface but also provides a low-impedance path for rapid, uniform charge transport along the microneedle, thereby improving the efficiency and stability of the triboelectric conversion process.

To further elucidate the electrical response under different mechanical inputs, the triboelectric behavior of LMS MN actuators with various stripe orientations was systematically examined during cyclic sliding (Fig. 5d and e). Under a fixed normal load, microneedle arrays oriented at 0°, 45°, and 90° relative to the sliding direction exhibit distinct voltage waveforms. In the 0° configuration, where the microneedles are largely aligned with the friction direction, the contact state is dominated by tangential sliding with relatively small periodic modulation of the effective contact area. The corresponding voltage amplitude is comparatively low, and the waveform shows small fluctuations associated with minor contact instabilities. At 45°, the inclined layout converts the imposed motion into a combination of sliding and intermittent normal modulation. This mixed friction mode improves charge separation and yields higher voltage amplitudes, together with a more regular and cleaner waveform. In the 90° configuration, the microneedles are perpendicular to the sliding direction and present the largest projected contact area, so that the interaction is dominated by combined normal and lateral friction. In this case, charge accumulation becomes more uniform, and the voltage output reaches the highest amplitude with a smooth, low-noise waveform, indicating a highly stable and reproducible charge transfer process from cycle to cycle (Fig. 5d). Variation of the sliding frequency further reveals the dynamic characteristics of the LMS MN triboelectric generator (Fig. 5e). As the friction frequency increases, the voltage waveform responds with a higher pulse repetition rate, effectively increasing the number of output cycles per unit time, while the peak voltage remains essentially stable over the tested range. This behavior suggests that the characteristic time for charge generation and recombination is much shorter than the mechanical period, enabling rapid electrical response without significant amplitude decay at higher excitation frequencies. The LMS MN system intrinsically mitigates humidity-induced performance degradation through the combined effects of BOPP encapsulation, a dense Spidroin matrix, and microneedle-enabled conformal contact, which collectively suppress moisture penetration, interfacial water accumulation, and charge leakage (Fig. S10). The results demonstrate that the LMS MN actuator can function as a geometry-tunable triboelectric generator, in which the LM-filled microneedle architecture and programmable stripe orientation jointly determine the magnitude, stability, and temporal profile of the electrical output.

### 2.5 The sensing performance and intelligent application of the LMS MN actuator

**Fig. 6:**
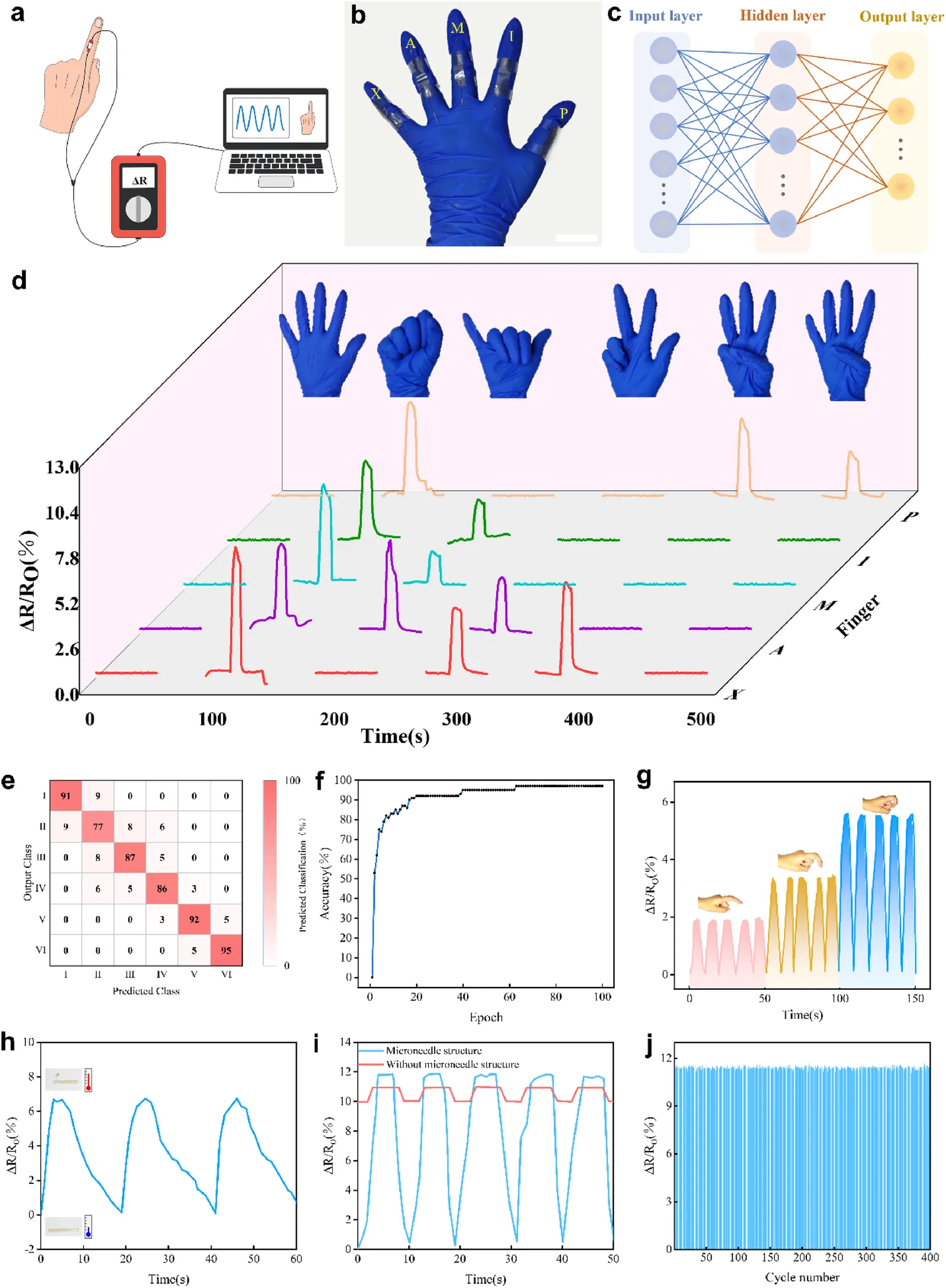
Machine-learning-optimized gesture recognition using LMS MN actuators. (a) Schematic of the LMS MN-based gesture sensing and recognition system. (b) Optical photograph of the instrumented smart glove. Scale bar: 3 cm.(c) Flowchart of the neural-network pipeline for multichannel sensor signals. (d) Six gestures and the corresponding five-finger ΔR/R_0_(%) waveforms. (e) Confusion matrix of classification results. (f) Recognition accuracy of the sensing-computing system versus training epoch. (g) Resistance change of the LMS MN actuators at finger-bending angles of 30°, 60°, and 90°.(h) Resistance modulation induced by LMS MN actuator-driven deformation. (i) Sensor responses with and without microneedles. (j) Stability of ΔR/R_0_ over 400 cycles of large deformation and recovery.

The perception and feedback of actuator deformation play crucial roles in intelligent soft systems. In the LMS MN actuator platform, deformation is not only generated on demand but also perceived and converted into electrical signals, enabling integrated actuation-sensing-computing functions. Fig. 6a outlines the workflow of a machine learning-assisted gesture recognition system composed of a soft sensing glove, an electrical signal acquisition module, and a computing unit. The structural configuration of the glove (Fig. 6b) features LMS MN strips conformally attached to the finger joints. When the fingers bend, the strips undergo synchronous stretching and compression, altering their internal LM conductive geometry and overall resistance. Each finger corresponds to an independent sensing channel. The recognition strategy (Fig. 6c) utilizes these multichannel ΔR/R_0_ signals as inputs to a neural network. The hidden layers automatically extract high-dimensional features from the time-dependent Δ R/R_0_ data, learning nonlinear mapping relationships between sensor signals and gesture labels. By training with sufficient data, this learning-based approach adaptively captures the intrinsic characteristics of different gestures, offering superior robustness over traditional rule-based methods. To demonstrate practical recognition, six representative gestures were defined and recorded in real time. As shown in Fig. 6d, different combinations of bent and straight fingers generate unique multilevel signal patterns across the five channels, forming a characteristic “electrical signature” for each gesture. The recognition performance is summarized in the confusion matrix (Fig. 6e), where strong diagonal values and low off-diagonal entries indicate that each predefined gesture is accurately distinguished. The evolution of recognition accuracy (Fig. 6f) shows a rapid increase in the initial training phase before approaching a stable plateau, confirming the model effectively learns the gesture mappings. To quantify single-actuator sensing performance, fixed-angle bending tests were conducted (Fig. 6g). The relative resistance change, Δ R/R_0_ = (R-R_0_)/R_0_, exhibits a clear positive correlation with the deformation angle. Well-separated plateaus are observed at 30°, 60°, and 90°, demonstrating the actuator can reliably distinguish multiple bending levels. Any device-to-device variations caused by manufacturing or LM distribution can be systematically calibrated against overall average behaviors. Fig. 6h investigates sensing behavior during photothermal actuation. Under near-infrared (NIR) illumination, local heating induces temperature-dependent bending, stretching the internal LM network. This deformation causes a rapid increase in Δ R/R_0_ that stabilizes when bending reaches a steady state, and gradually returns to baseline upon cooling. Because bending amplitude is governed by the local temperature field, the resistance change indirectly reflects thermal variations. This coupling provides a basis for temperature-sensitive self-sensing without separate thermal sensors, offering real-time feedback on the actuation process. The influence of microstructure is further examined (Fig. 6i) by comparing LMS strips with and without microneedle arrays. MN-integrated actuators show more pronounced Δ R/R_0_ responses and higher signal-to-noise ratios. This enhancement occurs because microneedles locally amplify stress and strain during bending, increasing the effective specific surface area and improving mechanical coupling with the LM conductive path, thereby validating their dual role in adhesion and sensing. Durability is crucial for long-term wearable and robotic applications. To assess fatigue resistance, the actuator was subjected to 400 cycles of significant bending and recovery (Fig. 6j). The Δ R/R_0_ waveform remains highly consistent without obvious baseline drift or amplitude attenuation, demonstrating that the LM network and microneedle architecture withstand repeated loading without structural failure. For prolonged real-world deployment, potential signal drift caused by material fatigue or sweat is addressed through a synergistic hardware-software strategy. Hardware-wise, moisture-resistant BOPP encapsulation and conformal microneedle-tissue interlocking intrinsically minimize sweat-induced interfacial fluctuations. Software-wise, dynamic baseline calibration or adaptive recurrent neural networks (RNNs) automatically correct drift, ensuring sustained recognition accuracy. Ultimately, the combination of internal LM functional fillers and microneedle microstructures enables LMS MN actuators to deliver significant directional deformation while simultaneously providing accurate sensing feedback, highlighting their suitability as multifunctional modules for next-generation human-machine interfaces.

### 2.6 Intelligent bandages based on LMS MN actuators for wound treatment

**Fig. 7:**
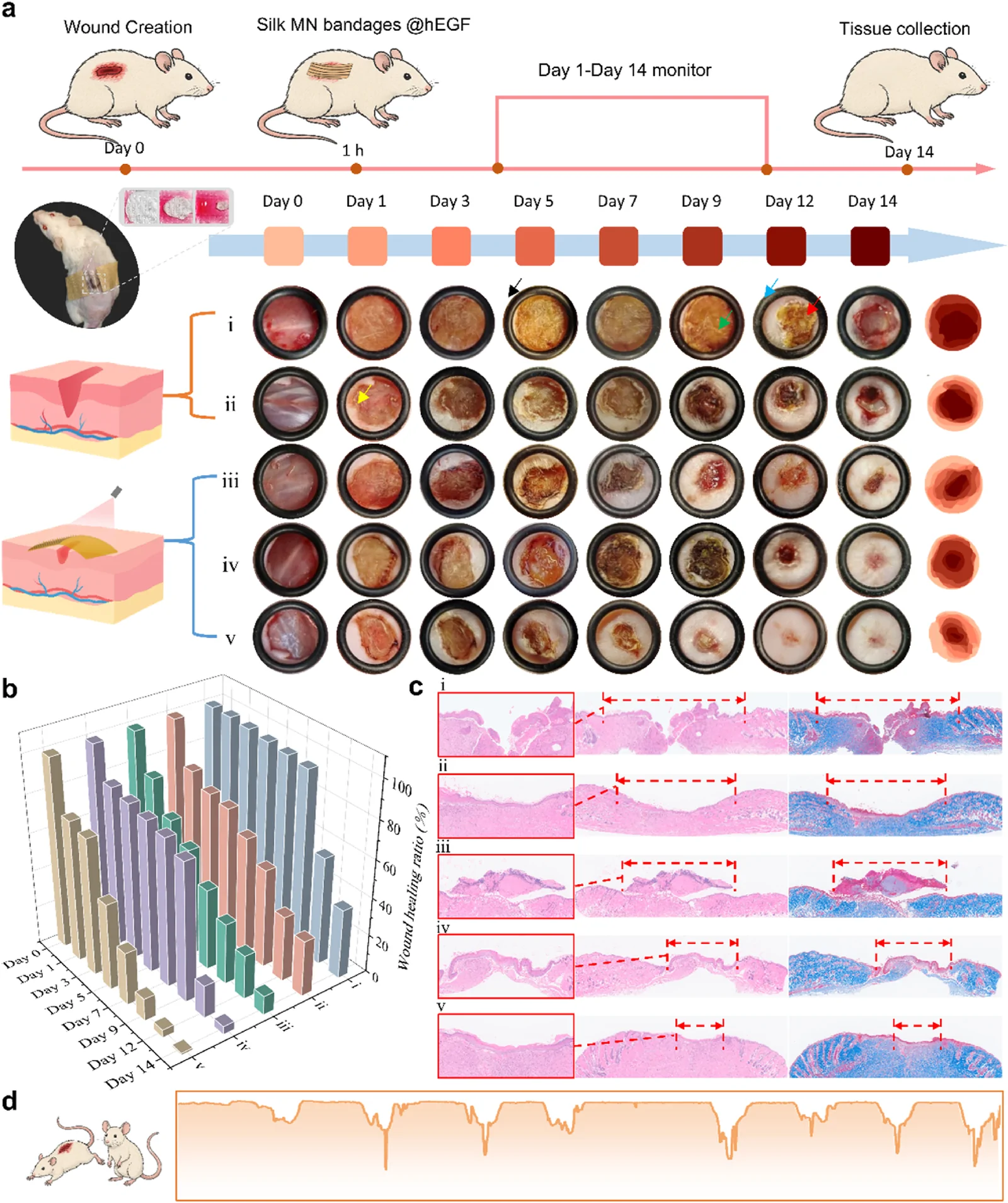
Treatment of epidermal incisions with intelligent bandages based on LM–Spidroin MN actuators. (a) Serial digital images of cutaneous wounds over Days 0, 1, 3, 5, 7, 9, 12, and 14 under five treatments: (i) control; (ii) only hEGF; (iii) blank Spidroin bandages; (iv) Spidroin microneedle (MN) bandages; (v) Spidroin MN bandages @hEGF. (Scale bar: 6 mm) (b) Quantification of the wound area (means ± SDs; n = 6).(c))Histological evaluation of wound healing in different groups via H&E and Masson staining; red arrows denote wound width. (scale bar: 6 mm) (d) By connecting electrodes, the mouse's movements are converted into visible electrical signals.

To explore the therapeutic potential of LMS microneedle (MN) actuators in a biologically relevant scenario, an intelligent Spidroin MN bandage was constructed and applied to an epidermal incision model. In this design, LMS MN actuators are embedded into a Spidroin-based bandage so that their programmed bending and microneedle penetration enable conformal wrapping of the wound surface and intimate contact with the tissue. The directional bending characteristics of the actuator allow the bandage to tighten around the defect, limiting wound edge retraction and maintaining a stable microenvironment, while the MN array enhances friction and local anchoring to reduce detachment risk. Live-cell fluorescence imaging visualized NIH-3T3 cells at the indicated culture time points, providing qualitative information on their morphology and distribution (Fig. S13). Cellular metabolic activity was assessed separately using the MTT assay after 1 and 3 days of treatment (Fig. S14). A full-thickness circular skin wound model was established on the dorsum of mice, and animals were randomly assigned to five treatment groups: untreated control(Fig. 7ai); only human epidermal growth factor (hEGF) (Fig. 7aii); blank Spidroin bandages(Fig. 7aiii); Spidroin MN bandages without drug(Fig. 7aiv); and Spidroin MN bandages loaded with hEGF (Spidroin MN bandages@hEGF) (Fig. 7av). Serial digital photographs of the wounds were captured on Days 0, 1, 3, 5, 7, 9, 12, and 14 to monitor the macroscopic healing process (Fig. 7a). Visual inspection indicates clear differences in wound closure among the five groups, the control wounds remain relatively large and irregular over the entire observation window; treatment with hEGF alone or with blank Spidroin bandages leads to moderate contraction but leaves noticeable residual defects at later time points. In contrast, wounds treated with Spidroin MN bandages show accelerated edge approximation, and those treated with Spidroin MN bandages@hEGF display the most rapid and homogeneous reduction in wound area, with nearly complete closure and smooth re-epithelialization by Day 14. These observations suggest that both the mechanical wrapping effect of the LMS MN bandage and the localized delivery of hEGF contribute synergistically to improved skin repair.

To quantify the macroscopic healing kinetics, the wound area in each group was measured from the serial images and normalized to the initial area, and the mean values (±SD, n = 6) were plotted as a function of time (Fig. 7b). The control group exhibits the slowest decrease in relative wound area throughout the 14-day period, reflecting the limited intrinsic healing capacity under untreated conditions. The hEGF-only and blank Spidroin bandage groups show somewhat faster contraction than the control, indicating that either biochemical stimulation or physical coverage alone provides partial benefit. Spidroin MN bandages without drug further improve the healing rate, consistent with the notion that directional deformation and mechanical confinement of the wound edges can reduce cracking and promote tissue approximation. The most pronounced effect is observed in the Spidroin MN bandages@hEGF group, where the wound area decreases rapidly from the earliest time points, and the closure ratio at each subsequent time is significantly higher than in the other four groups. By Day 14, the residual wound area in this group is minimal, indicating nearly complete closure. These quantitative data confirm that the combination of LMS MN-based intelligent bandaging and hEGF therapy substantially enhances wound healing efficiency compared with either factor alone.

Histological analyses provide further insight into the quality of tissue regeneration under different treatments (Fig. 7c). Hematoxylin and eosin (H&E) staining is used to evaluate granulation tissue formation, epidermal coverage, and general tissue morphology, while Masson staining highlights collagen deposition and dermal remodeling. In the control group, H&E sections reveal a wide, poorly bridged defect (indicated by red arrows), sparse granulation tissue, and incomplete re-epithelialization, in agreement with the slow macroscopic closure. The hEGF-only and blank Spidroin bandage groups show moderate improvements, with increased granulation tissue and partial narrowing of the wound gap, but the newly formed tissue remains relatively thin and disorganized. Samples treated with Spidroin MN bandages show a more continuous epithelial layer and denser underlying granulation tissue, suggesting that mechanical stabilization and enhanced microenvironment contribute to more effective tissue regeneration. The most advanced healing features are observed in the Spidroin MN bandages@hEGF group, H&E staining indicates a narrow wound width, well-developed granulation tissue, and an almost continuous epidermis spanning the original defect, while Masson staining reveals more abundant and better organized collagen fibers within the neodermis. These histological findings are consistent with the macroscopic and quantitative data, and confirm that intelligent bandages based on LMS MN actuators, when combined with hEGF loading, can both accelerate wound closure and improve the structural quality of the regenerated tissue. Overall, Fig. 7 demonstrates that LMS MN actuator-based intelligent bandages offer a promising strategy for active wound management, integrating programmable mechanical wrapping with localized biochemical therapy to achieve efficient and high-quality skin repair. It should be noted that the enhanced healing outcomes are driven by the synergistic integration of active programmable mechanical wrapping (which physically approximates the wound edges) and the sustained, continuous diffusion of hEGF from the structurally stable Spidroin matrix, rather than relying on a dynamically triggered or on-demand drug release mechanism. To analyze the interaction between the smart bandage and dynamic physiological states during the healing process, electrodes were connected to the LM–Spidroin MN system to monitor mechanical activity in real time (Fig. 7d). Mouse body movements were converted into corresponding electrical signals, demonstrating the feasibility of in vivo motor response-driven mechanisms. These results highlight the potential of LM–Spidroin MN-based devices as interactive wound dressings, capable of sensing and adapting to mechanical signals during the healing process. ICP-MS analysis showed that Ga remained below the method detection limit of 0.1 μg L^−1^ after the complete LMS MN actuator was immersed in PBS for 24 h, indicating no detectable Ga leaching during one intended dressing-application cycle (Table S4). Moreover, H&E staining revealed no evident treatment-associated histopathological abnormalities in the heart, liver, spleen, lung, or kidney of LMS MN-treated mice after 14 days (Fig. S18). Although there have been previous reports on smart wound dressings and soft robotic actuators, a comprehensive comparison of their mechanical toughness, photothermal response, triboelectric output, and wound healing efficacy shows that our LMS MN platform exhibits systemic superiority (Fig. S12a) [[37], [38], [39]]. Furthermore, compared with previously reported flexible hydrogels and E-skin elastomers, our LMS composite distinctly overcomes the traditional strength-ductility trade-off. It achieves an unprecedented tensile stress approaching 90 MPa with excellent elongation, demonstrating mechanical robustness that is vastly superior to other conventional clinical dressings (Fig. S12b).

## 3 Conclusion

To achieve multifunctional, biointegrated wound management, we developed a bioinspired liquid metal-spidroin (LMS) stripe microneedle actuator platform that bridges soft robotics and smart bandaging. The amorphous-region-modified spidroin matrix enables robust mechanical support, programmable stripe-induced deformation, and biocompatible microneedle anchoring, while the embedded gallium-based liquid metal droplets allow rapid photothermal actuation, highly conductive pathways, and efficient triboelectric charge collection. Moreover, the stripe-encoded microneedle geometry ensures morphology-programmed capillary microchannels for pump-free directional transport of biofluids and enhances deformation and temperature-sensitive resistance changes for self-powered sensing and gesture recognition. Comprehensive in vitro and in vivo studies demonstrated that LMS MN actuators achieve efficient light-driven crawling and rolling locomotion with high load-bearing capability, geometry-tunable triboelectric outputs and machine-learning-assisted motion decoding. Furthermore, in a mouse skin-injury model, the integrated intelligent bandages achieved significantly accelerated wound closure and improved re-epithelialization quality. Importantly, these enhanced healing outcomes are driven by the synergistic integration of passive physical protection, microneedle-mediated delivery of epidermal growth factor, and adjunctive photothermal-mechanical stimuli, rather than relying on a single isolated actuation mechanism. This LMS MN strategy, which leverages a unified materials platform and morphology-encoded architectures to combine actuation, adhesion, fluidics, and sensing, is expected to facilitate long-term conformal tissue integration and dynamic regulation of the wound microenvironment without bulky pumps or wired power supplies. Despite the promising multifunctional capabilities of the LMS MN actuators, several practical challenges must be addressed prior to clinical translation. First, the penetration depth of near-infrared light may be attenuated by thick, opaque wound exudates, potentially reducing the efficiency of photothermal actuation in heavily exuding wounds. Second, maintaining robust and conformal adhesion under extreme or continuous dynamic skin motion requires further optimization of the microneedle-tissue interface. Furthermore, the large-scale, cost-effective manufacturing of recombinant spidroin and the stringent regulatory approval pathways for medical devices incorporating liquid metals remain significant hurdles that must be carefully navigated in future studies. Therefore, the LMS stripe microneedle actuator system could serve as a promising tool for next-generation smart wound dressings, biointegrated soft robotic devices, and human–machine interfaces, and may inspire further studies in programmable, multifunctional actuators that seamlessly couple motion, fluid management, and self-powered health monitoring at the biological surface.

## 4 Experimental section

*Materials:* Silicone rubber liquids (Ecoflex) were purchased from SmoothOn, Inc., USA. Polyurethane (PU) 1624 was purchased from JITIAN Chemical Co., Ltd.(Shenzhen, China). The artificial Spidroin was composited in the authors’ laboratory (College of Pharmacy, Nanjing Industrial University). An advanced 30 kPSI cell crusher (T-S series, Constant Systems Limited) was employed during the cell lysis step to ensure the efficient release of the intracellular contents. All reagents used were not treated in any way. Luria-Bertani (LB) broth and agar were purchased from OXOID (USA). Ampicillin, imidazole, and isopropyl-β-D-thiogalactopyranoside (IPTG) were obtained from Sangon Biotech (Shanghai, China). The pSE-380 plasmid for molecular cloning was purchased from NovoPro Biotech Co., Ltd. (Shanghai, China), and oligonucleotide primers as well as DNA sequencing services were provided by Tsingke Biotech (Nanjing, China). Phanta Max Master Mix DNA polymerase, chemically competent *E. coli* BL21(DE3) cells, and a BCA protein assay kit were purchased from Vazyme Biotech Co., Ltd. (Nanjing, China). Restriction enzymes were obtained from Takara Bio (Beijing, China). Deionized water was used for the preparation of all aqueous solutions. Human epidermal growth factor (hEGF) was purchased from Beyotime Biotechnology (Nanjing, China). A eutectic gallium liquid metal was purchased from Shenyang Baijujie Co., Ltd. (Shenyang, China)

A total of 30 healthy SPF-grade male ICR mice (7-8 weeks old, weighing 30-35 g) were purchased from Reagan Biotechnology Co., Ltd. (Shanghai, China). All animal experiments were conducted in accordance with the Guide for the Care and Use of Laboratory Animals and were approved by the Human & Animal Investigation Ethics Committee of Nanjing Tech University (approval number: 20240711J). All experiments involving human subjects were approved by the Human & Animal Investigation Ethics Committee of Nanjing Tech University (approval number: 20240711J), and all participants provided informed, written consent.

*Preparation of Spidroin solution:*Recombinant Spidroin Expression and TSVT Purification: The recombinant spidroins used in this study were expressed in *E. coli* BL21 (DE3) cultured in Luria-Bertani (LB) liquid medium supplemented with 100 μg mL^−1^ ampicillin at 37 °C with 180 rpm agitation. When the optical density (OD) at 600 nm reached 0.6-0.8, IPTG was added to a final concentration of 0.3 mM to induce protein expression, after which the culture temperature was reduced to 30 °C and the cells were incubated at 180 rpm for an additional 24 h. At the end of the induction period, 300 mL of culture was harvested by centrifugation at 8000 × g for 10 min, and the supernatant was discarded. The resulting cell pellet was washed twice with 10 mM Na^+^-phosphate buffer (PBS, pH 7.5) and then resuspended in 30 mL of the same buffer. Cell disruption was performed by sonication at 400 W for 30-40 min, and the lysate was centrifuged at 12,000 × g for 10 min to separate the supernatant and precipitate. Because the expressed ARMs were predominantly present as insoluble inclusion bodies, the precipitated fraction was further purified using a two-step variable temperature (TSVT) strategy. The pellet containing ARMs inclusion bodies was mixed with an equal volume of deionized water in 50 mL centrifuge tubes and placed in a constant-temperature water bath. The temperature was first raised to 50 °C, and the suspension was gently stirred at 100 rpm for 20 min, after which the temperature was lowered to 20 °C and the mixture was incubated for 30 min. This temperature cycle (50 °C for 20 min followed by 20 °C for 30 min) was repeated a second time. After each complete cycle, the ARMs inclusion bodies were recovered by centrifugation. The overall TSVT procedure was carried out twice to obtain high-purity ARMs inclusion bodies, which were subsequently lyophilized and stored at −80 °C until further use [36].

*SDS-PAGE and Purity Analysis:* All the SDS-PAGE gels were 1.0 mm thick, with a 5% stacking gel at the top and a 12.5% separating gel at the bottom. The samples were prepared in 4× SDS-PAGE sample buffer (containing DTT), which included 2% SDS, 10% glycerol, 60 mM Tris (pH 6.8), 0.01% bromophenol blue, and 100 μM DTT. The samples were boiled at 95 °C for 5-8 min, followed by electrophoresis. The intensity of the target protein bands was analyzed relative to the sum of all protein bands in each lane via ImageJ software to calculate protein expression and purity.

*Preparation of the liquid metal-spidroin composite materials:* Recombinant spidroin was dissolved in HFIP to prepare a 20 wt% solution. LM was then incorporated at different weight fractions (0, 10, 25, 50, and 75 wt%, calculated relative to the combined dry mass of LM and spidroin) to obtain a series of spidroin–LM composite dispersions. Each mixture was ultrasonicated for 30 min and was removed from the ultrasonic bath at 10-min intervals for shaking for 5 min.

*Design of LMS MN Actuators:* LMS microneedle (MN) actuators were fabricated by combining a stripe-programmed mold with an amorphous region-modified Spidroin (ARM)/liquid metal (LM) casting ink. First, pressing commercial grating cards into room-temperature vulcanized silicone rubber (Ecoflex) yielded an elastic negative mold with parallel microgrooves. After curing and demolding, the mold was stretched to adjust groove spacing and laser-micromachined to open through-slots, generating the required stripe template. A hexafluoroisopropanol (HFIP) solution containing recombinant ARM and uniformly dispersed gallium-based LM microdroplets was cast into the mold to form a continuous protein-LM network. Following room-temperature solvent evaporation, a lightweight biaxially oriented polypropylene (BOPP) barrier film was laminated onto the surface. The demolded laminate was then uniaxially stretched to introduce prestrain and fix the in-plane bending axis. Subsequent laser micromachining patterned the stretched laminate into microneedle arrays and actuator strips. Finally, cutting the sheets at 0°, 45°, and 90° relative to the stripes yielded actuators with vertical, torsional, and curled deformation modes, respectively.

*Characterization:* All optical microscope images were captured with an optical microscope (SJ-U500, SAGA). SEM images were created by placing the sample on a conductive strip and coating it with a 20-nm gold layer using a Leica EM ACE600 high-vacuum sputtering coating (Leica Microsystems). The sample was then imaged using a scanning electron microscope (Field Electron and Ion Company, FEI). Images of HE and MT-stained sections were captured with an inverted biological microscope (Olympus BX63). HP-10 K HANDPI single-column materials tester to characterize the stress‒strain properties of inclined microneedles. Inclined microneedle hydrodynamics employ a plasma treatment device (CRF AP Plasma) to enhance the material surface through plasmaspraying technology. The generated output voltage was captured via a digital oscilloscope (DSO 01012A, Agilent Technologies).

Infrared Spectroscopy was performed to evaluate the secondary structure of the ARMs materials, particularly to assess the relative content of crystalline (β-sheet) and amorphous (random coil, α-helix, and β-turn) regions. This analysis enabled qualitative comparison of structural transitions between different ARMs variants. Thin films and were analyzed using infrared spectroscopy with a Thermo Scientific Nicolet S5 Fourier transform infrared (FTIR) spectrometer equipped with an iD5 ATR attachment. The spectra were initially processed via Origin 2021 software.

*Photothermal response performance testing of programmable flexible actuators:*The photothermal response of LMS microneedle (MN) actuators (typical size: 10 × 3 × 1.5 mm) under near-infrared irradiation was characterized using an 808 nm laser (power density: 2 W cm^−2^). During irradiation, the distance between the laser head and the actuator surface was fixed at 20 cm to ensure a stable and uniform illumination area. The deformation process of the LMS MN actuators was recorded in real time using a digital camera, and the evolution of bending curvature was obtained from the sequential images. Simultaneously, the temperature change and corresponding thermal images of the actuators were monitored by an infrared thermal imaging camera, allowing correlation of the photothermal temperature rise with the time-dependent mechanical response of the LMS MN actuators.

*Mechanical Tests:* Mechanical tests were carried out using an HP-10 K HANDPI single-column material testing machine to evaluate the uniaxial tensile properties of LMS MN actuators with different stripe orientations and backing layers. Rectangular LMS MN strips were prepared with the stripes oriented either perpendicular (90°) or parallel (0°) to the loading axis, and both bare actuators and BOPP-backed actuators were tested in each orientation. For each test, a single strip was clamped between the upper and lower fixtures of the tester with its long axis aligned with the loading direction. The crosshead was driven at a constant speed of 0.6 m min^−1^, and the force-displacement curves were recorded continuously until the specimen fractured or was completely pulled out of the grips [40].

*COMSOL Simulation:* Simulations were carried out using COMSOL Multiphysics to investigate capillary-driven fluid transport in patterned LMS channels and to evaluate the electrostatic potential distribution around a single LMS microneedle (MN) in the triboelectric configuration. For fluid transport, a 2D finite element model was established for straight LMS channels with stripe-only and stripe-plus-MN patterns, where the stripe/MN orientations were set to 0°, 45°, and 90° with respect to the main flow direction. The working fluid was treated as an incompressible Newtonian liquid governed by the continuity and Navier-Stokes equations

(1) ∇·u=0

(2) $$\rho \left(\frac{\partial u}{\partial t}+u\cdot \nabla u\right)=-\nabla p+\mu \nabla^{2}u\text{,}$$

where u is the velocity field, p is the pressure, ρ is the density, and μ is the dynamic viscosity. The channel walls were modeled as rigid, no-slip boundaries. Capillary-driven flow was introduced by imposing a pressure difference $\Delta p_{\text{cap}}$ between inlet and outlet corresponding to the effective capillary pressure of the patterned surface, approximated by the Young-Laplace relation

(3) $$\Delta p_{\text{cap}}=\frac{2\gamma \;\cos\theta }{r_{\text{eff}}}\text{,}$$

where γ is the surface tension, θ is the effective contact angle, and $r_{\text{eff}}$ is the effective curvature radius set by the stripe/MN geometry. The inlet pressure was fixed at $p_{\text{in}}=\Delta p_{\text{cap}}$ and the outlet pressure at $p_{\text{out}}=0$, while the lateral boundaries were treated as symmetry planes or solid walls depending on the specific pattern. Steady-state solutions were used to extract velocity fields, pressure distributions, and streamlines, which were analyzed to quantify the influence of stripe/MN orientation on flow directionality, local hydraulic resistance, and average transport velocity.

For the triboelectric case, a coupled electrostatic model was constructed to evaluate the potential distribution on a single LMS MN during contact-separation. The MN was modeled with the actual LMS geometry, consisting of a conductive LM core encapsulated by a dielectric shell, facing a flat dielectric counter surface with opposite triboelectric polarity. The electrostatic field was described by Poisson's equation

(4) $$\nabla \cdot \left(\varepsilon \nabla \phi \right)=-\rho_{f}\text{,}$$

where ϕ is the electric potential, ε is the permittivity, and $\rho_{f}$ is the free charge density derived from the imposed surface charge densities σ on the friction interfaces. The LM core was set as an equipotential electrode ($\phi =\phi_{\text{MN}}$), and the counter surface was grounded (ϕ=0). For a given separation distance d, the effective potential difference V(d) between the MN electrode and ground was obtained from the simulated field as

(5) $$V\left(d\right)=\phi_{\text{MN}}\left(d\right)-\phi_{\text{ground}}\text{.}$$

All simulations were solved using time-dependent or parametric sweep solvers with adaptive meshing. This ensured accurate resolution of steep velocity and electric-field gradients, enabling quantitative comparison with the experimental measurements.

*In Vivo Wound Healing Experiment:* A total of 30 healthy SPF-grade male ICR mice (7-8 weeks old, weighing 30-35 g) were used in the in vivo wound-healing experiment. After anesthesia, a full-thickness circular skin wound approximately 1 cm in diameter was created on the dorsal region of each mouse using a sterile scalpel. The mice were then randomly allocated to five treatment groups, with six mice in each group (n = 6 mice per group): the untreated control group, the hEGF-only group (topical hEGF application only), the blank Spidroin bandage group (Spidroin bandages without drug), the Spidroin microneedle (MN) bandage group (drug-free Spidroin MN bandages), and the Spidroin MN bandage@hEGF group (Spidroin MN bandages loaded with hEGF). Each mouse constituted one independent biological replicate. Prior to application, all experimental dressings were sterilized via ultraviolet (UV) irradiation in a biosafety cabinet for 30 min per side. This non-thermal surface sterilization method was specifically utilized to prevent heat-induced denaturation of the spidroin matrix and to maintain the stability of the liquid metal network. To ensure proper skin penetration and close contact, the corresponding dressing was applied to the wound and gently pressed for 2 min. All experimental dressings were replaced every 24 h. After 14 days of treatment, granulation tissue samples were collected from the wound bed and fixed in neutral buffered formaldehyde. The tissues were subsequently dehydrated, embedded in paraffin, and sectioned into 5 μm slices for histological evaluation using Masson's trichrome (MT) staining and hematoxylin and eosin (H&E) staining. To minimize observation bias, these histological sections were independently evaluated by researchers blinded to the group assignments.

*Cell Culture and Biocompatibility Assessment:* NIH-3T3 cells were first cultured in a humidified incubator (37 °C and 5% CO2, Waltham 150i, Thermo Scientific). The cells were cultured in DMEM supplemented with 10% fetal bovine serum (FBS) and 1% penicillin‒streptomycin. When the cell density reached the desired level, NIH-3T3 cells were inoculated onto the culture surface of the osmotic solution from the LMS MN actuator. Cell growth was observed (after 1 and 3 days). Live-cell fluorescence staining was used to visualize NIH-3T3 cell morphology and distribution, and images were acquired using a fluorescence microscope. Cellular metabolic activity was evaluated separately using the MTT assay. The MTT experiments were performed in 96-well plates in three independent experiments, with six technical replicate wells per treatment group in each experiment. Cell viability was calculated using the formula: Cell viability = (ODtreat – ODblank)/(OD0 – ODblank) ×100%, where ODtreat is the absorbance of the treated group, ODblank is the absorbance of the blank (medium only), and OD0 is the absorbance of the untreated control group.

*Statistical Analysis:* All the calculations were performed at a sample size of n = 6. One-way analysis of variance (ANOVA) followed by Tukey's post-hoc test was used to examine the statistical significance among the multiple compared groups. “p < 0.05” was selected as the significance level, and “*” indicates “0.01 < p < 0.05”, “**” indicates “p < 0.01”, and *** indicates “p < 0.001”. The processed data were presented as the mean values with their corresponding standard deviations. Origin 2021 was used for the statistical analysis.

## CRediT authorship contribution statement

**Xin Li:** Writing – original draft, Visualization, Methodology, Investigation, Formal analysis. **Min Xue:** Investigation. **Kaiwen Zhou:** Investigation. **Ran Xu:** Software. **Hao Ren:** Writing – review & editing. **Bingbing Gao:** Writing – review & editing, Resources, Funding acquisition. **Chwee Teck Lim:** Writing – review & editing. **Feng Liu:** Writing – review & editing.

## Declaration of competing interest

The authors declare that they have no known competing financial interests or personal relationships that could have appeared to influence the work reported in this paper.

## Data Availability

Data will be made available on request.
