# Supplementary material for: Bioinspired liquid metal-spidroin (LMS) stripe microneedle actuators
Source: Mater Today Bio. 2026 Sep 10;40:103662. doi: 10.1016/j.mtbio.2026.103662 (PMC13586579; doi:10.1016/j.mtbio.2026.103662)
Supplement: Supplementary file 1 [file mmc1.docx]

Supporting Information

**Bioinspired Liquid Metal-Spidroin (LMS) Stripe Microneedle Actuators**

*Xin Li*^a^ *, Min Xue*^a^ *,* *Kaiwen Zhou*^a^*, Ran Xu*^a^ *,Hao Ren*^a^*, Bingbing Gao*^a,b,^**,* Chwee Teck Lim ^b,c,d^ *and Feng Liu*^e,^***

^a^ College of Biotechnology and Pharmaceutical Engineering, School of Pharmaceutical Sciences, Nanjing Tech University, Nanjing 211816, China.

^b^ Institute for Health Innovation and Technology (iHealthtech), National University of Singapore, Singapore 117599, Singapore.

^c^ Department of Biomedical Engineering, National University of Singapore, 117583, Singapore.

^d^ Mechanobiology Institute, National University of Singapore, 117411, Singapore.

^e^ Research Center of Allergy & Immunology, Shenzhen University School of Medicine, Shenzhen, China.

* Correspondence: [gaobb@njtech.edu.cn](mailto:gaobb@njtech.edu.cn); [fliuout@outlook.com](mailto:fliuout@outlook.com)

**Figure S1.** Physical schematic diagram of the grating card mould.

**Figure S2.** The ecoflex uniaxially stretched by 1, 1.2, 1.3, and 1.5 times (i) is subsequently laser-engraved (ii), ultrasonically cleaned (iii), and formed into a microneedle mould with strips (iv). Following PU moulding, this facilitates observation of the final product's form.

**Figure S3.** The ecoflex undergoes bidirectional stretching at ratios of 1, 1.2, 1.3, and 1.5 times (i), followed by laser engraving (ii) and ultrasonic cleaning (iii). This process produces a microneedle mould with stripes (iv), which is then cast in polyurethane (PU) to facilitate observation of the final product's form.

**Figure S4.** Scanning Electron Microscope image of the stripe section of the LMS MN actuator

**Figure S5.** The morphology of ARM dissolved in hexafluoro-2-propanol (HFIP) and after the addition of liquid metal (LM), along with corresponding physical images of the resulting films.

**Figure S6.** Raman spectral image of the LMS MN actuator

**Figure S7.** Comparative COMSOL simulations of pumpless fluid transport in patterned LMS channels evaluating the effect of fluid viscosity. (a) Simulated velocity fields and pressure distributions for a baseline fluid. (b) Simulated velocity fields and pressure distributions for a viscous fluid mimicking human wound interstitial fluid (viscosity = 1.5*10-3 Pa·s). The results demonstrate that the morphology-encoded capillary microchannels maintain reliable transport efficiency and directional flow even under clinically relevant viscous conditions.

**Figure S8.** BOPP Adhesion Test.

**Figure S9.** Bar chart quantifying wound area (mean ± standard deviation; n=6)

|  | i | ii | iii | iv | v |
| --- | --- | --- | --- | --- | --- |
| Day 0 | 100 | 100 | 100 | 100 | 100 |
| Day 1 | 99.93905 | 78.54133 | 81.49333 | 84.0163 | 74.38092 |
| Day 3 | 98.97405 | 72.54593 | 66.12355 | 81.9075 | 72.13147 |
| Day 5 | 97.7043 | 70.97825 | 57.17476 | 79.90055 | 45.50758 |
| Day 7 | 96.70882 | 61.80817 | 42.96736 | 77.87563 | 27.81667 |
| Day 9 | 95.48479 | 52.08181 | 32.32831 | 73.0889 | 11.7853 |
| Day 12 | 57.13342 | 34.74181 | 23.39475 | 17.20585 | 4.096979 |
| Day 14 | 36.92925 | 29.58926 | 10.04518 | 4.283489 | 1.01128 |

**Table S1.** Charts quantify wound area (%) (mean ± standard deviation; n=6)

|  | Day 0 | Day 1 | Day 3 | Day 5 | Day 7 | Day 9 | Day 12 | Day 14 |
| --- | --- | --- | --- | --- | --- | --- | --- | --- |
| i | 19689 | 19677 | 19487 | 19237 | 19041 | 18800 | 11249 | 7271 |
| ii | 17797 | 13978 | 12911 | 12632 | 11000 | 9269 | 6183 | 5266 |
| iii | 19701 | 16055 | 13027 | 11264 | 8465 | 6369 | 4609 | 1979 |
| iv | 16692 | 14024 | 13672 | 13337 | 12999 | 12200 | 2872 | 715 |
| v | 15426 | 11474 | 11127 | 7020 | 4291 | 1818 | 632 | 156 |

**Table S2.** Charts quantify wound area (pixels) (mean ± standard deviation; n=6)

**Figure S10.** Electrical output performance of the triboelectric nanogenerator based on the LMS MN actuator under different humidity conditions. The output signal voltage was measured under relative humidity conditions of 30%, 60%, and 90% RH.

**Figure S11.** Photothermal actuation performance of LMS MN actuators. (a) Schematic and optical images of reversible NIR-triggered deformation. (b) Time-resolved infrared images during NIR irradiation. (c) Bending angle as a function of temperature. (d) Load-to-actuator-weight ratio during deformation. (e) Repeated bending and recovery under NIR ON/OFF cycles. (f) Bending and recovery kinetics under different NIR power densities.

**Figure S12.** (a)Comprehensive comparison of mechanical toughness, actuation sensitivity, triboelectric output, wound closure rate, and multifunctional integration between state-of-the-art smart devices reported in other studies and the LMS MN actuators fabricated in this study. (b)Comparison of different flexible materials and conventional clinical dressings in terms of tensile strength and elongation at break.

**Figure S13.** Fluorescence images of live-stained NIH-3T3 cells on days 0, 1, and 3, showing cell morphology and distribution. Scale bar: 100 μm.

**Figure S14.** Relative metabolic activity of NIH-3T3 cells evaluated by the MTT assay. The assay was performed in three independent experiments, n = 6 A *p* value < 0.05 was considered statistically significant. *p* < 0.001 (***).

**Figure S15.** Optimization of the spidroin/LM composition. (a) Photographs of spidroin–LM composite dispersions with different LM contents. (b) Stress–displacement curves of samples containing 0, 10, 25, and 50 wt.% LM. The 75 wt.% LM sample was excluded from mechanical testing because of insufficient structural integrity.

**Figure S16.** Histological evaluation of wound repair in the different treatment groups on day 14. a Representative overview and enlarged H&E-stained sections and Masson’s trichrome-stained sections of wounds treated with (i) no treatment (control), (ii) hEGF only, (iii) a blank Spidroin bandage, (iv) a drug-free Spidroin microneedle (MN) bandage, or (v) an hEGF-loaded Spidroin MN bandage. The rows are arranged from groups i to v from top to bottom. Enlarged H&E views corresponding to the regions indicated in the overview sections are shown in the left column. Red dashed double-headed arrows delineate the residual wound region, while blue staining in the Masson sections indicates collagen deposition. b Representative high-magnification H&E images of the five groups, arranged as groups i–iii in the upper row and groups iv–v in the lower row. E, epidermal alteration or re-epithelialization; F, fibroblast-rich granulation tissue; V, newly formed vessel; I, inflammatory-cell infiltration; H, focal hemorrhage; X, inflammatory exudate or protein deposition. The hEGF-loaded Spidroin MN bandage group exhibited the narrowest residual wound region, relatively continuous epithelial coverage, and dense collagen deposition, supporting the most advanced overall tissue repair among the five groups. Scale bars: 100 μm in the enlarged/high-magnification H&E images.

| **Treatment group** | **Residual wound area on day 14 (%)** | **Wound closure on day 14 (%)** | **Representative high-magnification H&E findings** | **Histological injury score** | **Integrated interpretation** |
| --- | --- | --- | --- | --- | --- |
| Control | 36.93 | 63.07 | Granulation tissue with fibroblast proliferation, sparse newly formed vessels, limited inflammatory-cell infiltration, focal hemorrhage, and residual epidermal necrosis and detachment | 3, moderate | The slowest wound closure, accompanied by incomplete epidermal repair and persistent moderate tissue injury |
| hEGF only | 29.59 | 70.41 | Epithelial coverage with epidermal and keratin thickening, fibroblast-rich granulation tissue, sparse newly formed vessels, and limited inflammatory-cell infiltration | 2, mild | hEGF alone moderately improved epithelial repair but produced only a limited improvement in macroscopic wound closure |
| Blank Spidroin bandage | 10.05 | 89.95 | Extensive epidermal degeneration and necrosis, abundant surface inflammatory exudation and protein deposition, limited fibroblast proliferation, occasional newly formed vessels, and focal inflammatory-cell infiltration | 3, moderate | Macroscopic wound contraction was improved, but microscopic tissue maturation remained incomplete and inflammatory exudation was still evident |
| Spidroin MN bandage | 4.28 | 95.72 | Epithelial coverage with epidermal and keratin thickening, fibroblast-rich granulation tissue, sparse newly formed vessels, limited inflammatory-cell infiltration, and focal mild hemorrhage | 2, mild | Near-complete wound closure with improved epithelial continuity and mild residual histological injury |
| **Spidroin MN bandage@hEGF** | **1.01** | **98.99** | Epithelial coverage with epidermal and keratin thickening, fibroblast-rich granulation tissue, sparse newly formed vessels, limited inflammatory-cell infiltration, and focal mild hemorrhage | **2, mild** | **The most complete macroscopic wound closure, together with mild residual histological injury and the more abundant and better organized collagen observed in Figure 7c, indicates the most advanced overall tissue repair among the five groups** |

**Table S3.** Integrated assessment of macroscopic wound closure and histopathological repair on day 14.

**Figure S17.** SEM-EDS characterization of the liquid metal-spider silk protein (LMS) composite. (a) SEM image of the analysis area. (b) Overlay of EDS elemental distribution maps. (c-f) Corresponding elemental distribution maps of C (c), N (d), O (e), and Ga (f). The uniform Ga elemental signal indicates that the liquid metal droplets are uniformly distributed in the spidroin matrix. All elemental distribution maps are taken from the same region. Scale bar: 10 μm.

**Figure S18.** H&E staining of major organs from the control and LMS MN-treated mice. Scale bars: 200 μm.

| **Sample** | **Element** | **Exposure time** | **Number of extracts** | **Instrumental measurements** | **Method detection limit (μg L⁻¹)** | **Result** |
| --- | --- | --- | --- | --- | --- | --- |
| PBS extract of the LMS MN actuator | Ga | 24 h | 1 | 3 | 0.1 | ND (<0.1 μg L⁻¹) |

**Table S4.** ICP-MS analysis of Ga leaching from the LMS MN actuator after immersion in PBS for 24 h.

**Figure S19.** Skin penetration performance of the LMS microneedles. (a) Representative optical images of the skin surface (i) before and (ii) after application of the LMS microneedle patch, showing visible micropuncture sites. (b) Representative H&E-stained cross-sectional image of the treated skin, confirming that the microneedles penetrated through the epidermis into the superficial dermis to a depth of approximately 400 μm. Scale bar: 400 μm.

**Figure S20.** Triboelectric output performance and operational stability of the device. (a) Long-term output-voltage stability during 12,000 s of repeated operation; the insets show representative waveforms at the beginning and end of the test. (b) Output voltage and current as functions of external load resistance. (c) Corresponding power density versus load resistance. (d) Short-circuit current density and transferred charge density under different loading forces.

**Figure 21.** Bioinformatic characterization of the Amy-ab-1 sequence design. (a)Sequence-level annotation of Amy-ab-1 highlights the distribution of FGAILSS amyloid-forming motifs, charged residues and glycine-rich flexible positions across the 85-residue core sequence. (b)Amino-acid composition and computed physicochemical descriptors summarize glycine enrichment, charged-residue content, predicted isoelectric point and hydropathy. (c)IUPred3 predictions indicate a predominantly flexible sequence landscape. (d)ESMFold-derived acidic-to-basic residue distances assess potential ionic interactions, but should be interpreted cautiously because of the low mean model confidence.(e) Sliding-window beta-sheet and amyloid-zipper proxy scores identify FGAILSS-associated segments with elevated structural propensity. (f)A coarse-grained assembly proxy visualizes transient motif-centred contacts. (g)The integrated scorecard and linkage map summarize how sequence-derived features connect to proposed functional domains including mechanics, actuation, sensing and repair.
